# Corn oil-formulated *Cordyceps javanica*: a high-performance, low-impact tool for integrated tick management

**DOI:** 10.1007/s10493-026-01117-y

**Published:** 2026-03-02

**Authors:** Joana da Rocha Matos, Adriani da Silva Carneiro, Thaís Almeida Corrêa, Emily Mesquita, Laura Nobrega Meirelles, Victória Silvestre Bório, Américo de Castro Monteiro-Sobrinho, Tadeu Augusto van Tol de Castro, Andrés Calderín García, Mariana Guedes Camargo, Isabele da Costa Angelo, José Francisco Arruda e Silva, Eliane Dias Quintela, Patrícia Silva Gôlo, Vânia Rita Elias Pinheiro Bittencourt

**Affiliations:** 1 Postgraduate Program in Veterinary Sciences, Veterinary Institute, Federal Rural University of Rio de Janeiro, RJ Seropédica, Brazil; 2 Laboratory of Soil Biological Chemistry, Department of Soils, Institute of Agronomy, Federal Rural University of Rio de Janeiro, Seropédica, RJ Brazil; 3 University of Vassouras, Vassouras, RJ Brazil; 4 Department of Epidemiology and Public Health, Veterinary Institute, Federal Rural University of Rio de Janeiro, Seropédica, RJ Brazil; 5 Laboratory of Entomology, EMBRAPA Rice and Beans, GO Santo Antônio de Goiás, Brazil; 6 Department of Animal Parasitology, Veterinary Institute, Federal Rural University of Rio de Janeiro, Seropédica, RJ Brazil

**Keywords:** Biological control, Entomopathogenic fungus, Southern cattle tick, Sustainable tick control

## Abstract

Mycoacaricides represent a promising alternative for managing resistant tick populations. This study evaluated the efficacy of corn oil–based *Cordyceps javanica* formulations against non-parasitic stages the southern cattle tick *Rhipicephalus microplus* under laboratory and semi-field conditions. Additionally, fungal persistence in soil and possible alterations in soil composition were assessed using ATR-FTIR spectroscopy combined with chemometric analysis. Corn oil (1%, 3%, and 5%) combined with 0.01% silicone oil did not affect conidial germination (> 98.4%). Corn oil and *C. javanica* suspensions alone yielded larval average mortality of 20.1% and 18.8%, respectively, 15 days after the treatment. When formulated in corn oil, averages of larval mortality ranging from 64.1% on day 5 to 100% on day 15, indicating a synergistic effect between fungus and oil. Corn oil alone resulted in a female tick control percent ranged from 20.5 to 73.3%, while the fungus alone achieved 28.2% and 14.9% control at 10⁷ and 10⁸ conidia/mL, respectively. All fungus–oil formulations significantly reduced female reproductive efficiency, achieving 90–100% tick control. Under semi-field conditions, the 10⁸ conidia/mL + 3% corn oil formulation reduced larval recovery from *Urochloa decumbens* pots by 98.6% compared to the oil and silicone control. Thirty days after application, *C. javanica* persisted in soil at 2.1 ± 0.3 × 10⁵ CFU/g (39.4% recovery). ATR-FTIR analysis showed no detectable alterations in the soil’s chemical profile following application of any formulation. These results highlight the potential of oil-based *C. javanica* formulations as an effective and sustainable tool for integrated tick management in livestock systems.

## Introduction

*Rhipicephalus microplus* is an ectoparasite of great importance in livestock farming, especially for cattle, due to the damage caused to meat and dairy productivity and the potential for transmission of pathogens (da Silva et al. 2013; Klafke et al. 2024). Currently, *R. microplus* control is performed by synthetic acaricides, however, the improper use of this method has led to the selection of resistant tick populations (Klafke et al. 2024), including multiple resistance to different classes of acaricides (Reck et al. 2014; Dzemo et al. 2022). This scenario drives the search for sustainable control alternatives.

Entomopathogenic fungi such as *Beauveria* spp. and *Metarhizium* spp. have been studied as alternatives for tick control under both laboratory and field conditions (Beys-da-Silva et al. 2020; Camargo et al. 2014; 2016; Meirelles et al. 2023; Mesquita et al. 2020; Marciano et al. 2021; Castro-Saines et al. 2024). These fungi act as natural control agents by infecting and colonizing the host, leading to the parasite’s death and reducing the tick population. Recent studies demonstrate the high efficacy of oil-based formulations with these fungi (Barbieri et al. 2023). Some authors discuss the oil action on the cuticular adhesion of the fungus, which may contribute to its effectiveness and persistence on tick surfaces (Camargo et al. 2012; Marciano et al. 2020, 2021; Mesquita et al. 2020).

Another entomopathogenic fungus, *Cordyceps* spp., formerly known as *Isaria*, has been shown potential as a biological agent for tick control (Angelo et al. 2012; Wang et al. 2023). Lee et al. (2022) screened various *Cordyceps* isolates against the tick *Haemaphysalis longicornis* and found that *Cordyceps militaris* produced bioactive compounds with antiparasitic properties. Similarly, *Cordyceps* sp. has demonstrated significant virulence against *R. microplus*, with conidia formulations effectively reducing tick larval densities under both laboratory and field conditions (Angelo et al. 2012; Wang et al. 2023). This is supported by the findings of Lopes et al. (2023), who investigated the diversity of anamorphic *Cordyceps* spp. from Brazilian agricultural sites, highlighting the fungi’s broader biocontrol potential.

Several studies have investigated the use of oil-based formulations combined with entomopathogenic fungi for tick control (Nogueira et al. 2020; Camargo et al. 2016; Muniz et al. 2020; Marques et al. 2019). One of the main purposes of these formulations is to protect fungal conidia from abiotic stressors such as solar radiation, high temperatures, and desiccation, thereby enhancing their persistence and efficacy under field conditions (Murillo-Alonso et al. 2023). Additionally, the effectiveness of fungal conidia in adhering to the arthropods’ cuticle increased with oily adjuvants (Beys-da-silva et al. 2020; Nogueira et al. 2020). Kim et al. (2011) formulated *Cordyceps fumosorosea* conidia using corn oil, demonstrating improved long-term storage stability and enabling its use in the biological control of the whitefly *Trialeurodes vaporariorum.* In addition to these adjuvants, silicone oil has also been used for herbicides, insecticides, and acaricides as it improves the spreadability of active components, also impacting the physiology of arthropods by promoting asphyxiation (Cowles et al. 2000; Chen et al. 2022). A field trial by Barbieri et al. (2023) demonstrated that *Metarhizium anisopliae* mineral oil-based formulations (2.5% mineral oil + 0.01% silicone oil) effectively reduced *R. microplus* infestations in cattle; one of the formulations achieved up to 66% efficacy by day 21. Although these adjuvants enhanced fungal performance, it is equally important to assess their potential environmental impact. Oil residues, particularly those derived from mineral sources, may alter soil structure and microbial activity (Tamada et al. 2012; Klamerus-Iwan et al. 2015; Polyak et al. 2023). On the other hand, vegetable oils can offer advantages over mineral oils due to their renewable and biodegradable nature, which can reduce long-term environmental impact and ensure a more sustainable approach (Siniawski et al. 2007; Rosado-Aguilar et al. 2017; Lee et al. 2022).

The present study aimed to test the compatibility of different formulations based on corn vegetable oil and silicone oil with the fungus *C. javanica*, as well as their efficacy in controlling the non-parasitic stages of the tick *R. microplus* under laboratory and semi-field conditions. Additionally, the persistence of the fungus in the soil was assessed. ATR-FTIR spectroscopy combined with chemometric techniques was also employed to detect the presence of silicone and to investigate potential alterations in the soil profile resulting from the field application of the different formulations. The *C. javanica* isolate BRM 27,666 used in our study originates from the commercial product Lalguard^®^ C99, which was registered for the control of the whitefly *Bemisia tabaci* in 2022, following seven years of collaborative research between Embrapa and Lallemand Plant Care Brazil (Patos de Minas, MG) (BRASIL 2022). This product has demonstrated high efficacy in managing whitefly populations across commercial cropping areas in all regions of Brazil (Boaventura et al. 2021, 2025).

## Materials and methods

### Rhipicephalus microplus ticks

*Rhipicephalus microplus* ticks used in the experiments originated from the Laboratory of Microbial Control of Arthropods of Veterinary Importance of Federal Rural University of Rio de Janeiro (UFRRJ). Tick larvae (Porto Alegre strain, known to be susceptible to synthetic acaricides) were used to artificially infest calves, in accordance with the protocol approved by the Ethics Committee on the Use of Animals (CEUA) of the Veterinary Institute (IV) of UFRRJ, Brazil (protocol number 9714220419). The animals were housed in dedicated pens at the Experimental Station for Parasitological Researchers, Wilhem Otto Neitz (EEPPWON) of UFRRJ. Fully engorged females were collected directly from the pen floors 21 days post-infestation, washed with tap water, and then immersed in a 0.05% sodium hypochlorite solution for three minutes. After disinfection, the females were dried and divided into groups with homogeneous weights for the corresponding treatments. Part of the engorged females was used directly in bioassays with adult ticks, while the remainder was maintained to obtain larvae from their oviposition, which were subsequently used in separate larval bioassays.

### Fungal strain

The *Cordyceps javanica* isolate BRM 27666 was obtained from *Bemisia tabaci* nymphs infected in soybean fields in Porangatu, Goiás, Central Brazil. The isolate is preserved in the Invertebrate Fungal Collection at Embrapa Rice and Beans, Santo Antônio de Goiás, Brazil, and was identified as *C. javanica* by Lopes et al. (2023).

Aerial conidia of *C. javanica* were suspended in sterile distilled water containing 0.01% (v/v) polyoxyethylene sorbitan monooleate (Tween 80^®^, Vetec Fine Chemicals LTDA, Brazil). The fungal concentration was adjusted to 1 × 10^8^ conidia/mL using a hemocytometer. Before the assay, conidial viability was determined by plating a 10 µL aliquot of a 1 × 10^5^ conidia/mL suspension on potato dextrose agar medium (PDA) and incubating at 25 ± 1 °C and relative humidity (RH) ≥ 80%. Germination of propagules was determined 24 h after incubation. An aliquot of 10 mL of fungal suspension was inoculated into spawn bags with 1 kg of rice and 300 mL of 0.5% pre-autoclaved peptone solution (Santi et al. 2011). The rice bags were stored at 25 ± 1 °C and RH ≥ 80% for 21 days. The fully colonized substrates were then washed with sterile distilled water containing 1% Tween 80, sieved, and used in the experiments.

### Effect of corn and silicone oils on *Cordyceps javanica* conidial germination

Formulations were prepared by sequentially adding the following components: 1% Tween 80 aqueous solution, fungal conidia (1 × 10⁵ conidia/mL), 1%, 3%, or 5% corn oil, and 0.01% silicone oil (Silicone^®^, Loja Synth, Brazil). Formulations were incubated for one hour to assess whether pre-application exposure to corn oil and silicone oil could compromise fungal viability.

To assess fungal viability in the formulations, the oil phase of the emulsions was removed prior to inoculation onto PDA using 100 µL of Solub’Oil (General Chemicals^®^, Brazil), according to Paixão et al. (2017). The suspensions were prepared according to the percentage of corn oil and a fixed concentration of silicone oil, all with a final volume of 2 mL. Conidia were washed with an aqueous Solub’Oil solution and then transferred to a 15 mL centrifuge tube. Samples were vigorously shaken for 2 min and centrifuged (Daiki DT-4500, IonLab, Brazil) at 1578 *g* for 5 min. The supernatant was discarded, and conidia were resuspended in 1% (v/v) Tween 80^®^ aqueous solution. Fungal germination was assessed by plating 20 µL of each conidial suspension of each treatment on PDA supplemented with 0.002% (w/v) benomyl. The low concentration of benomyl prevents excessive growth of the germ tube without negatively affecting the germination process, allowing the counting of germinated and non-germinated conidia. The plates were then incubated at 25 ± 1 °C and RH ≥ 95%. Conidial germination was evaluated by microscopy (400×; E200, Nikon^®^, Japan) 24 h and 48 h post-incubation. All treatments were tested in triplicate, and the experiment was repeated three times.

### In vitro bioassay with *Rhipicephalus microplus* larvae

The virulence bioassay with tick larvae was conducted according to Camargo et al. (2012). Ten glass test tubes (15 mL each) were used per group, containing approximately 1000 larvae (50 mg of *R. microplus* eggs) in each tube. The eggs were previously incubated at 27 °C ± 1 °C and RH ≥ 80% for 15 days until larval hatching. The tubes with less than 95% hatchability were discarded. The treatments were arranged in thirteen groups, as follows: *Controls* – two groups (Control 1–1% aqueous suspension of Tween 80; Control 2 – emulsion containing 0.01% silicone and 1% Tween 80), *Oil-only groups* – three groups (Corn oil at 1, 3 and 5% emulsified with 1% Tween 80 and 0.01% silicone oil); *Fungus-only groups* – two groups (*C. javanica* at 1 × 10⁷, and 10⁸ conidia/mL, each prepared in a 1% aqueous suspension of Tween 80). *Fungus + oil groups* – six groups (*C. javanica* at 1 × 10^7^ and 10^8^ conidia/mL combined with corn oil at 1%, 3%, and 5%, all emulsified with 1% Tween 80 and 0.01% silicone oil). Each group of larvae was immersed in 1 mL of each treatment for 3 min. The test tubes were then inverted to remove excess conidial suspension/formulation, allowing absorption through a cotton plug. All treatments were incubated at 25 ± 1 °C and RH ≥ 80%. Larval mortality was assessed every five days for 15 days. Each treatment was performed in triplicate, and the entire experiment was repeated three times using new conidial suspensions each time.

### In vitro bioassay with *Rhipicephalus microplus* engorged females

*Rhipicephalus microplus* engorged females were weighed individually and divided into homogeneous weight classes (all groups had females weighing from 0.1400 to 0.2150 g). The treatments were similar to those described above for larvae, except for control 2, which consisted of an emulsion containing 0.01% silicone and 1% Tween 80. For each treatment, 10 engorged tick females were used, with each tick considered an independent experimental unit. The ticks were individually submerged for three minutes in 1 mL of each treatment. Then, each female was placed in Petri dishes (90 × 15 mm) and incubated at 25 ± 1 °C and RH ≥ 80%. The egg mass of each female was collected, weighed, stored individually in a 10 mL injectable glass vial, and incubated at 25 ± 1 °C and RH ≥ 80%. The evaluated parameters included larval hatching percentage, egg production index (EPI), nutritional index (NI) (Bennett 1974), and the percentage of tick control (CP). The reproductive efficiency (RE) was determined to calculate CP, with EPI and NI derived from the ratio of egg mass to initial weight of engorged females, and CP calculated as the reduction in RE of treated groups compared to controls (Drummond et al. 1971; Camargo et al. 2012). Tick females were considered dead when they failed to respond to stimulation, presented a darkened or shriveled cuticle, and/or exhibited visible mycelial growth on the body surface. The bioassay was repeated three times, using new conidial suspensions each time.

### Semi-field bioassay with *Rhipicephalus microplus* females

Fifty plastic pots (22 cm height and 24 cm width) were filled with a substrate consisting of one-third sand and gravel for water drainage and two-thirds non-sterile soil conditioner composed of peat, crushed pine bark, wood sawdust, vermiculture, rock dust, decomposed plant, and forest organic matter (Natussolos do Brasil LTDA^®^, Brazil).

Commercial *Urochloa decumbens* seeds Stapf (Poales: Poaceae) cv. Marandu (Wolf^®^ Seeds do Brasil, Brazil) encrusted with antifungal coating formulation (Maxim XL, Fludioxonil Metalaxyl-M) (Hochst. Ex A. Rich.) were sown into soil conditioner. The pots were placed in an outdoor area of the EEPPWON of UFRRJ (Seropédica, RJ, Brazil; 22°44′S and 43°42′W) from May to October 2024. They remained exposed to direct sunlight until 3:00 p.m., receiving partial shade for the rest of the day. The plants were grown for 110 days prior to treatment with daily irrigation and regular monitoring to ensure uniformity. According to the Brazilian National Institute of Meteorology, during the experimental period (post-treatment), weekly mean temperatures ranged from 24.1 °C to 27.2 °C, and weekly mean relative humidity ranged from 57% to 75%.

Based on the *in vitro* results of the bioassay with larvae and engorged females of *R. microplus*, the 3% corn-oil formulation was selected for the semi-field test. Five groups with ten pots each were tested: (I) Control 1–1% aqueous suspension of Tween 80, (II) Control 2 – emulsion of 0.01% silicone oil and 1% Tween 80, (III) 3% Corn oil in 1% Tween 80, (IV) 3% Corn oil in 1% Tween 80, and 0.01% silicone oil, (V) Fungus at 1 × 10^8^ conidia/mL + 3% corn oil in 0.01% silicone oil in 1% Tween 80. For treatment application, 100 mL of each formulation was applied using a small low-volume hand sprayer (1.2 L Pre-Compression Spray Nozzle, Guarany^®^; Brazil) designed to ensure homogeneous coverage over the entire soil surface. The fungal concentration used in group V was equivalent to 2.21 × 10^7^ conidia/cm^2^. On the same day, after the application, three completely engorged females, with homogeneous weight, were placed on each of the 50 grass pots. The pots were surveyed daily for female oviposition and larval hatching. The larvae were collected from the tops of the *U. decumbens* leaves and counted starting 38 days after the fungal treatment. The leaves were cut, and larvae were collected from the cut grass material, frozen and counted. The pots were then surveyed daily until no more larvae were observed.

### Persistence of *Cordyceps javanica* in the treated soil

Soil samples were collected from each pot (Controls [I and II] or treated [III, IV and V]) at three time points: collected at 24 h before treatment, 24 h after, and 30 days after treatment. Soil was collected with a spatula from three random points in each pot to a depth of no more than 5 cm and combined into a single homogeneous sample. A portion of soil (0.35 ± 0.05 g) place it in a 1.5 mL microtube with 1 mL of 0.01% Tween 80 sterile distilled water solution. Fifty microliters of this homogenized suspension were plated on CTC medium (Fernandes et al. 2010) (PDA supplemented with 0.25 g/L of cycloheximide, 0.01 g/L of thiabendazole, 0.05 g/L of chloramphenicol), using a Drigalski handle (one plate per pot per date). The number of *C. javanica* colony forming units (CFUs) per gram was determined based on the number of *C. javanica* colonies observed in each Petri dish, considering samples from all 10 pots. Colonies of *C. javanica* were re-isolated from each plate, transferred to PDA medium, and identified based on colony morphology, color, background, and conidial shape (Humber 2012).

### Analysis of soil samples by attenuated total reflection fourier transform infrared spectroscopy (ATR – FTIR)

Soil samples from the *U. decumbens* pots (50 g from each pot) were collected from each treated pot (Aqueous control - I; Silicone oil control - II; Oil control 3% - III; Oil control 3% and silicone oil - IV; and Fungus formulated - V) and homogenized (final weight of ~ 500 g per treatment). Thirty soil samples were analyzed considering: five treatment groups, three dates of sampling (24 h before the treatment, 24 h and 30 days after the treatment), and two replicates per treatment. The soil samples (= 250 g) were freeze-dried (LIOTOP L101, Series 62215, Liobras^®^, Brazil) for 10 days. One gram of each lyophilized sample was used for the analysis.

The spectra were recorded in the 4.00 to 4.000 cm^− 1^ region at a 4 cm^− 1^ resolution. For this purpose, a (VERTEX 70/70v FTIR spectrometer, Bruker Corporation^®^, Germany) was coupled to a platinum diamond attenuated total reflection device (ATR), in which the diamond disk functioned as an internal reflection element. The lyophilized soil samples were placed in the ATR crystal to record the spectra. An air spectrum was recorded and used as a blank before each analysis. The spectra were collected at a room temperature of approximately 25 °C. The spectra were collected using OPUS-Bruker software. The spectral work was performed using the ACD/Labs Software v.2020.1.1. A total of 38 ATR-FTIR spectra were obtained, comprising 30 soil samples from previously described treatments, six samples consisting of two replicates of commercial standard silicone added to soil from aqueous control group (1:1; MIX) from tree different times (collected at 24 h before treatment, 24 h after and 30 days after treatment), and two reference spectra acquired directly from the pure silicone standard (Sigma-Aldrich^®^,USA ). All the spectra were subjected to baseline correction, and a Savitzky–Golay smoothing algorithm was applied to increase the signal-to-noise ratio. Characteristic bands were identified by plotting the peak positions with the PeakPicking tool (Prieto García et al. 2016).

### Chemometric analyses of ATR-FTIR spectral data

The ATR-FTIR spectra loadings generated a 38 ⋅ 3997 matrix of samples. The ATR-FTIR matrix was displayed as a line plot for visual inspection and subsequently transformed through “normalization”, “smoothing-Savitsky-Golay”, and baseline correction (that is, an offset-linear baseline correction). The transformed matrix was plotted using the matrix color tool.

The average spectra were obtained using the descriptive statistics tool and plotted in line form after accessing the results matrix. The matrix corresponding to the average spectra for each soil type was then added to the original matrix to perform a multivariate data analysis. The standardized matrix was used to perform a principal component analysis (PCA). A total of seven components and automatic outlier detection were used in the PCA model. A nonlinear iterative partial least squares (NIPALS) algorithm and cross-validation were applied. The scores and loadings were graphed separately, where the loadings were plotted in line form to visualize the weights as a spectral pattern.

### Statistical analysis

Data were checked for normality using a Shapiro–Wilk test. A 4-sample test for equality of proportions without continuity correction was conducted to compare the germination rates across the different groups (fungal suspension without oil, and 1%, 3%, and 5% corn oil fungal formulations) at both time points (24 h and 48 h after incubation). The in vitro bioassay with larvae and engorged females, and the analysis of adult ticks’ survival had non-normal distributions and were analyzed with Kruskal–Wallis followed by Dunn’s test in GraphPad Prism version 8.4.2 for Windows (GraphPad Software, USA). Mortality curves for *R. microplus* females were adjusted according to a nonlinear Weibull model and compared using the Chi-square test (*P* < 0.05). This nonparametric statistical method was used for the comparison of two unpaired groups to verify whether or not they belong to the same population, and when the requirements for the application of the Student’s t-test were not met. To estimate the median lethal time (LT_50_), the Weibull model was fitted, and values were compared by the overlapping of their 95% confidence intervals (95% CI) using the package “drc 3.0–1”. The LT_50_ was not estimated for treatments where mortality did not reach 50%.

Larval recovery from the bioassay under semi-field conditions and the persistence of *C. javanica* in the soil had normal distributions. CFU persistence of *C. javanica* data were analyzed using one-way ANOVA followed by a t-test (LSD). Larval recovery data were analyzed by one-way ANOVA followed by Tukey’s test using GraphPad Prism 8.4.2., P-values < 0.05 were considered significant.

The combinations (different oil concentrations plus fungus) were considered synergistic if the calculated χ^2^ value was greater than 3.84 and the observed mortality was higher than the expected mortality. The treatment was considered antagonistic if the calculated χ^2^ value for any combination exceeded 3.84, but the observed mortality was lower than expected. Combinations with a calculated χ^2^ value less than 3.84 were categorized as having no interaction effect (Et-tazy et al. 2025).

## Results

### Effect of corn oil and silicone on *Cordyceps javanica* conidial germination

Conidial germination averages of *C. javanica*, 24 h after incubation, reached 99.8 ± 0.24% in the control and 99.3 ± 0.22%, 98.4 ± 0.22%, and 98.8 ± 0.17% in the 1%, 3%, and 5% corn oil treatments, respectively. No significant differences were observed among treatments at this time (χ² = 3.81, df = 3, *P* = 0.282). At 48 h after incubation, germination rates were 99.96 ± 0.11% (control and 1% corn oil), 99.59 ± 0.54% (3% corn oil), and 99.67 ± 0.28% (5% corn oil), with χ² = 1.13 (df = 3, *P* = 0.771). Therefore, corn oil, had no effect on conidial germination, and fungal viability remained stable across all concentrations.

### In vitro bioassay with *Rhipicephalus microplus* larvae

Larval mortality (%) in the control groups (1% Tween 80 and 0.01% Silicone oil + 1% Tween 80) was below 1.2%, exhibiting significantly lower values than all other treatments at 5, 10 and 15 days after treatment (Table 1). Corn oil, at all tested concentrations, resulted in less than 20.1% mortality: however, larval mortality increased with higher corn oil concentrations across all evaluation dates (Table 1). *C. javanica* suspension at 1 × 10⁸ conidia/mL caused significantly higher larval mortality than the lower concentration (1 × 10⁷ conidia/mL) on all three evaluation dates; however, mortality remained ≤ 18.8% (Table 1). When *C. javanica* was formulated in corn oil, *R. microplus* larval mortality ranged from 64.1% on day 5 to 100.0% on day 15, significantly enhancing the fungal efficacy (Table 1). All treatments with the formulated fungus differed significantly from the other treatments, and a synergistic effect was observed for all fungus–corn oil combinations (Table 2).

**Table 1:** Mean mortality (%) ± standard deviation of *Rhipicephalus microplus* larvae treated with different concentrations of corn oil and *Cordyceps javanica* at 10^7^ and 10^8^ conidia/mL alone and formulated with corn oil

Treatments^1^	5 days	10 days	15 days
Control 1 (Tween 80 1%)	0.67 ± 1.7 ^i^	1.17 ± 3.1 ^h^	1.16 ± 3.1 ^g^
Control 2 (Silicone 0.01% + Tween 80 1%)	0.50 ± 1.5 ^i^	0.83 ± 2.6 ^h^	1.0 ± 2.7 ^g^
Corn oil 1%	2.8 ± 4.1 ^d, h^	4.5 ± 5.9 ^g^	4.83 ± 6.3 ^f^
Corn oil 3%	10.5 ± 6.2 ^g^	13.1 ± 6.6 ^f^	4.17 ± 6.9 ^e^
Corn oil 5%	14.0 ± 5.8 ^f^	16.8 ± 5.16 ^e^	20.1 ± 4.2 ^d^
10^7^ conidia/mL			
Fungus	9.0 ± 3.5 ^g^	12.33 ± 3.6 ^f^	16.6 ± 2.7 ^e^
Fungus + corn oil 1%	64.1 ± 7.8 ^e^	74.8 ± 6.7 ^d^	95.3 ± 7.4 ^c^
Fungus + corn oil 3%	88.1 ± 9.6 ^b^	94.6 ± 6.8 ^b^	99.1 ± 3.7 ^a, b^
Fungus + corn oil 5%	81.3 ± 14.1 ^c^	92.5 ± 9.8 ^b^	100% ^a^
10^8^ conidia/mL			
Fungus	12.8 ± 4.4 ^f^	16 ± 3.8 ^e^	18.8 ± 3.8 ^d^
Fungus + corn oil 1%	72.1 ± 8.2 ^d^	87.1 ± 7.2 ^c^	98.5 ± 4.1 ^b^
Fungus + corn oil 3%	89.3 ± 9.1 ^b^	98 ± 3.8 ^a^	100 ^a^
Fungus + corn oil 5%	95.1 ± 6.2 ^a^	99.5 ± 2.7 ^a^	100 ^a^

^1^Emulsions of corn oil in Tween 80 at 1%, and silicone at 0.01%; fungal suspensions in 1% Tween 80; Conidia of the fungus formulated in corn oil at 1%, 3% and 5%) and silicone (0.01%) in Tween 80 at 1%. Means with the same letter in the same column (days) do not differ significantly considering *P* < 0.05 (Kruskal–Wallis test)

**Table 2:** Interactions between *Cordyceps javanica* and corn oil +silicone oil based on observed mortality to *Rhipicephalus microplus* larvae 15 days after the treatment

Combined treatment	Observed mortality (OM)	Expected mortality (EM)^a^	χ² calc^b^	Type of interaction^c^
10^7^ conidia/mL + corn oil 1%	95.33	20.69	269.17	Synergistic
10^7^ conidia/mL + corn oil 3%	99.17	28.48	175.48	Synergistic
10^7^ conidia/mL + corn oil 5%	100	33.48	132.18	Synergistic
10^8^ conidia/mL + corn oil 1%	98.50	22.75	252.21	Synergistic
10^8^ conidia/mL + corn oil 3%	100	30.33	160.02	Synergistic
10^8^ conidia/mL + corn oil 5%	100	35.20	119.28	Synergistic

^a^Expected mortality EM = Mf + Mi (1-Mf/100), with Mf and Mi being the observed per cent mortalities caused by the fungus and the oil respectively. ^b^Chi-squared values calculated by the equation: χ² = (OM-EM)^2^ /EM, and then compared to the chi-squared table value for *df* = 1 at a = 0.05 (χ² ) table = 3.84). ^c^Additive effect: χ² calcs 3.84, synergistic effect: χ² calc ˃ 3.84 and (OM-EM) >0, and no effect: χ² calc ≤ 3.84 and EM OM

### In vitro bioassay with *Rhipicephalus microplus* engorged females

All concentrations of corn oil significantly reduced *R. microplus* egg production compared to the control group (Table 3). Although *C. javanica* alone, at 10⁷ and 10⁸ conidia/mL, did not affect EPI values, its formulation in corn oil, regardless of the oil concentration, resulted in a significant reduction in EPI in comparison to the control (Table 3). NI values were similar to the control for corn oil at 1% and the fungus at both concentrations (Table 3). As observed for the EPI, the oil-based formulation of the fungus significantly reduced the NI values. Corn oil concentrations reduced RE compared to the control, and the reduction increased with higher oil concentrations. Corn oil alone controlled 20.5% to 73.3% of tick females. The fungal suspension at 1 × 10⁷ conidia/mL significantly reduced RE compared to the control, whereas 1 × 10⁸ conidia/mL exhibited no effect. Female control by *C. javanica* alone reached 28.2% and 14.9% at 1 × 10⁷ and 10⁸ conidia/mL, respectively. In contrast, all formulations combining the fungus with corn oil caused a significant reduction in RE, achieving 90% to 100% female control (Table 3). The survival analysis revealed significant differences among treatments (Table 4). Comparison of survival curves by the chi-square test (*P* ≤ 0.05) showed that the oil-based fungal formulations (both concentrations of 1 × 10⁷ and 10⁸ conidia/mL) differed significantly from the control and from the individual components (fungus or oil alone). The most pronounced effects were observed for the formulations with 3% and 5% corn oil, which were statistically distinct from all other treatments (*P* < 0.001).

**Table 3:** Biological parameters of *Rhipicephalus microplus* engorged females immersed in different concentrations of corn oil, *Cordyceps javanica* at 10^7^ and 10^8^ conidia/mL alone and formulated with corn oil

Treatments^1^	Egg production index (%)	Nutrient index (%)	Reproductive efficiency (%)	Tick Control (%)
Control 1 (Tween 80 1%)	49.4 ± 11.4 ^a^ (26)	74.5 ± 16.0 ^a^ (26)	49.3 ± 11.3 ^a^ (26)	-
Corn oil 1%	39.2 ± 17.0 ^b^ (26)	66.6 ± 27.8 ^a^ (26)	39.2 ± 17.0 ^b^ (26)	20.5
Corn oil 3%	30.2 ± 25.4 ^c^ (26)	49.6 ± 42.3 ^b^ (26)	29.7 ± 24.7 ^c^ (26)	39.8
Corn oil 5%	26.0 ± 21.2 ^c^ (26)	48.1 ± 35.9 ^b^ (26)	13.2 ± 16.4 ^d^ (26)	73.3
Dose 10^7^ conidia/mL				
Fungus	43.6 ± 16.5 ^a, b^ (26)	70.4 ± 22.2 ^a^ (26)	35.1 ± 20.4 ^b^ (26)	28.2
Fungus + corn oil 1% (n)	14.2 ± 15.9 ^d^ (26)	29.6 ± 27.0 ^c^ (26)	4.9 ± 10.6 ^e^ (26)	90.0
Fungus + corn oil 3% (n)	1.6 ± 7.5 ^e^ (26)	4.5 ± 21.4 ^d^ (26)	0.60 ± 3.0 ^f^ (26)	98.8
Fungus + corn oil 5% (n)	1.7 ± 8.7 ^e^ (26)	3.5 ± 17.7 ^d^ (26)	1.7 ± 8.7 ^f^ (26)	96.5
Dose 10^8^ conidia/mL				
Fungus	43.1 ± 17.2 ^a, b^ (26)	66.2 ± 22.9 ^a^ (26)	42.0 ± 17.9 ^a, b^ (26)	14.9
Fungus + corn oil 1% (n)	15.0 ± 15.5 ^d^ (26)	30.7 ± 29.3 ^c^ (26)	5.0 ± 12.3 ^e^ (26)	89.8
Fungus + corn oil 3% (n)	3.3 ± 10.0 ^e^ (26)	6.7 ± 20.0 ^d^ (26)	0.90 ± 3.6 ^f^ (26)	98.2
Fungus + corn oil 5% (n)	0.13 ± 0.68 ^e^ (26)	0.45 ± 2.29 ^d^ (26)	0.0 ± 0 ^f^ (26)	100.0

^1^Emulsion of corn oil in Tween 80 at 1%, and silicone at 0.01%; fungal suspension at 10^7^ and 10^8^ conidia/mL in 1% Tween 80; Fungus conidia formulated in corn oil (1%, 3% or 5%) and silicone (0.01%) in Tween 80 at 1%. Means with the same letter in the same column do not differ significantly considering *P* < 0.05 (Kruskal–Wallis test). (*n* = 26) indicates the total number of ticks used for each treatment, considering all replicates

**Table 4:** P-values from the statistical comparisons of mortality curves of *Rhipicephalus microplus* females following treatment with *Cordyceps javanica* at 10⁷ and 10⁸ conidia/mL, formulated with corn oil at 1% and 3%

Treatment	Control 1	10^7^	10^8^	Oil 1%	Oil 3%	Oil 5%	10^7^+oil 1%	10^8^+oil 1%	10^7^+oil 3%	10^8^+oil 3%	10^7^+oil 5%
10^7^	0.1628781	.	.	.	.	.	.	.	.	.	.
10^8^	0.2797920	0.7360822	.	.	.	.	.	.	.	.	.
Oil 1%	0.7094769	0.3000662	0.4757022	.	.	.	.	.	.	.	.
Oil 3%	0.0319738	0.3407520	0.2117092	0.0659200	.	.	.	.	.	.	.
Oil 5%	0.0161139	0.2908246	0.1643542	0.0387841	0.6344308	.	.	.	.	.	.
10^7^+oil 1%	0.0000002	0.0000947	0.0002260	0.0000090	0.0018125	0.0043635	.	.	.	.	.
10^8^+oil 1%	0.0000001	0.0000001	0.0000001	0.0000001	0.0000001	0.0000001	0.0001508	.	.	.	.
10^7^+oil 3%	0.0000001	0.0000001	0.0000001	0.0000001	0.0000001	0.0000001	0.0000960	0.7432392	.	.	.
10^8^+oil 3%	0.0000010	0.0000570	0.0001340	0.0000060	0.0014983	0.0029380	0.5968979	0.0005934	0.0004314	.	.
10^7^+oil 5%	0.0000001	0.0000001	0.0000001	0.0000001	0.0000001	0.0000001	0.0024964	0.4180466	0.3028961	0.0069312	.
10^8^+oil 5%	0.0000001	0.0000001	0.0000001	0.0000001	0.0000001	0.0000001	0.0000854	0.4396484	0.5413735	0.0003558	0.2056625

Mortality considered significant different at P *<* 0.05. Control 1 at 1% aqueous suspension of Tween 80; Oil at 1, 3 and 5% emulsified with 1% Tween 80 and 0.01% silicone; 10⁷, and 10⁸ conidia/mL, all prepared in a 1% aqueous suspension of Tween 80. 10^7^ and 10^8^ conidia/mL mixed with corn oil at 1%, 3%, and 5%, all emulsified with 1% Tween 80 and 0.01% silicone

**Fig. 1:**
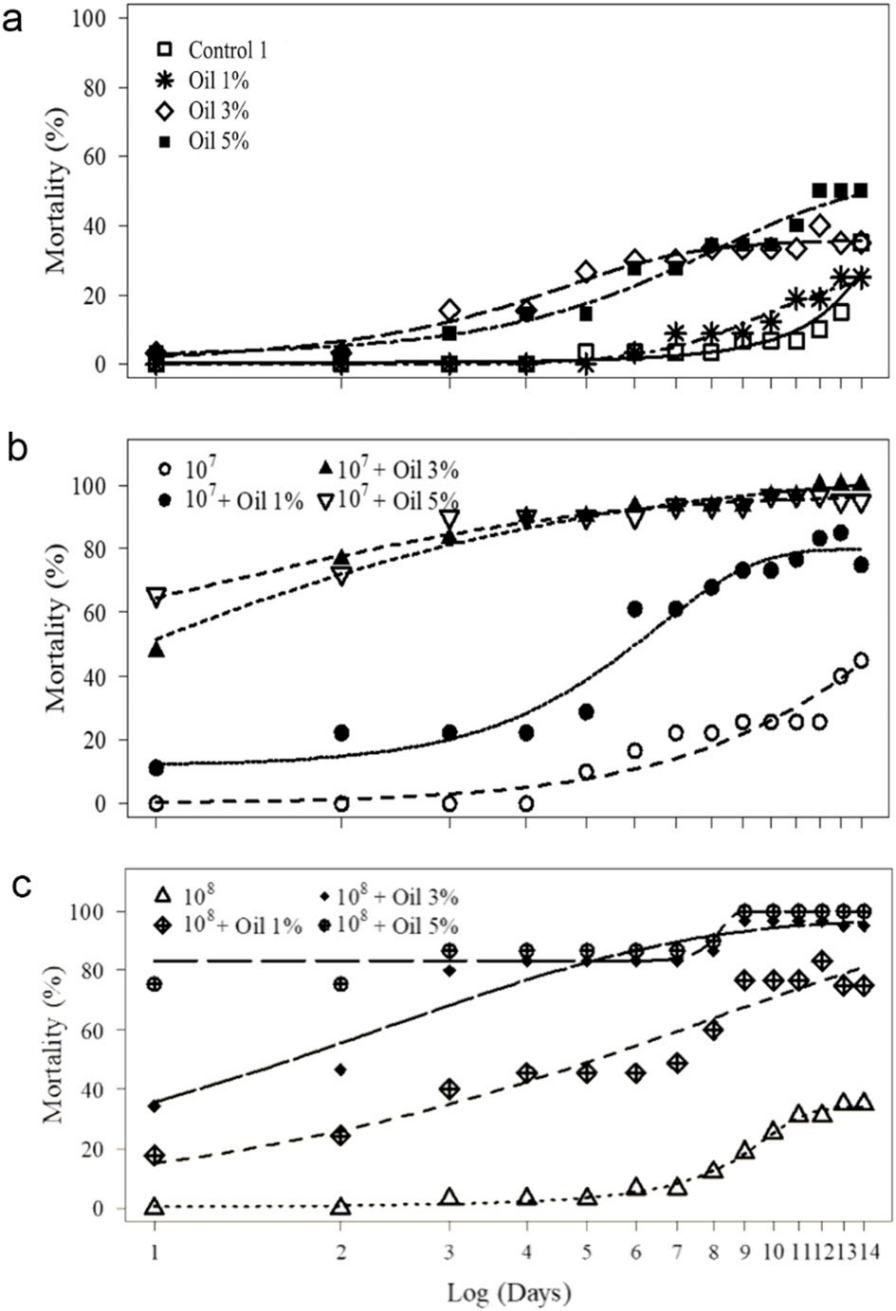
Mean mortalities at different days post-inoculation for *Rhipicephalus microplus* engorged females immersed in corn oil at (**a**) 1, 3, 5% and (**b**) *Cordyceps javanica* at 10^7^ and 10^8^ conidia/mL alone and (**c**) formulated with corn oil at 1, 3, and 5%. Curves were adjusted according to non-linear model Weibull

Formulations combining *C. javanica* with corn oil at 1% and 3% promoted a rapid decline in tick female survival, reaching 100% mortality within the first few days after immersion, whereas females treated with *C. javanica* alone or with corn oil alone exhibited a slower mortality pattern, similar to that of the control (Fig. 1). Estimated median lethal times (LT₅₀) confirmed the high virulence of the formulations, with the lowest LT₅₀ values for *C. javanica* + 3% corn oil: 0.97 days (10⁷ conidia/mL) and 1.6 days (10⁸ conidia/mL) (Table 5). Treatments with corn oil alone at 5% or with the fungus unformulated presented higher LT₅₀ values (> 14 days), indicating reduced pathogenicity. The synergistic effect between *C. javanica* suspensions and the corn oil was evident, as all formulated treatments reduced LT_50_ by more than 85% compared to the unformulated fungus (Table 5).

**Table 5:** Estimates of parameters of non-linear model Weibull and median lethal time (LT_50_) of *Rhipicephalus microplus* females after treatment with *Cordyceps javanica* at 10^7^ and 10^8^ conidia/mL formulated with corn oil at 1, 3, and 5%

Treatments	Weibull Model parameters a				LT 50 (LC 95%)
	b	e	c	d	
Control 1 (Tween 80 1%)	2.96017	-0.00114	1	22.58284	-
10^7^	1.85269	-0.00491	1	18.8735	-
10^8^	2.23161	-0.00228	1	18.69446	-
Oil 1%	2.74318	-0.00208	1	20.59023	-
Oil 3%	0.87327	-0.00117	1	26.87371	-
Oil 5%	1.30031	-0.00038	1	17.31871	14.1 (14.01 - 14.27)
10^7^ conidia/mL + corn oil 1%	1.20268	-0.00085	1	8.02941	6.02 (5.2 - 6.7)
10^7^ conidia/mL + corn oil 3%	0.65949	-0.00116	1	1.41464	0.97 (0.78 - 1.16)
10^7^ conidia/mL + corn oil 5%	0.28971	-1.80722	1	0.07847	-
10^8^ conidia/mL + corn oil 1%	0.87337	-0.00032	1	7.83144	5.1 (4.6 - 5.5)
10^8^ conidia/mL + corn oil 3%	0.77368	-0.0014	1	2.5497	1.6 (1.3 - 1.9)
10^8^ conidia/mL + corn oil 5%	36.80722	0.83472	1	8.15028	-

^a^ Model parameters: b = B is the slope factor around the “e” parameter; c = is the lowest asymrate of the curve; d = is the upper asymrate of the curve; e = is the inflection point of the curve

### Semi-field bioassay with *Rhipicephalus microplus* females

**Fig. 2:**
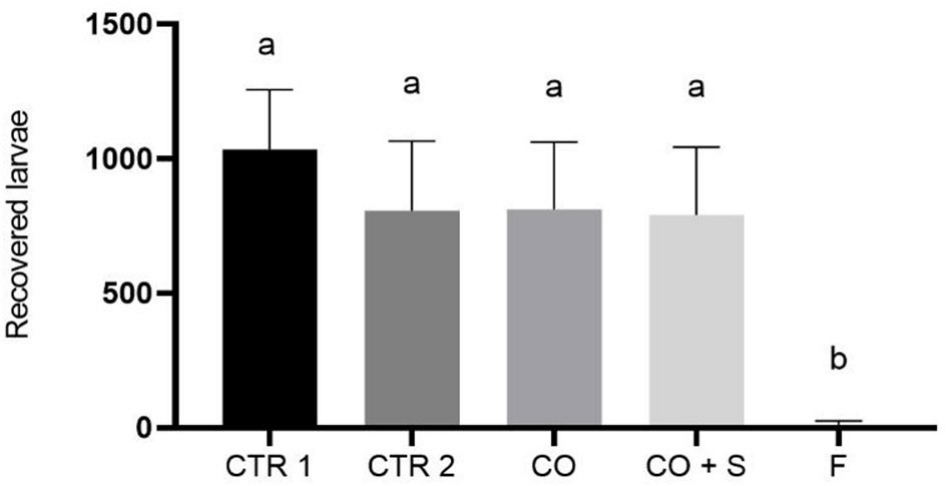
Mean and standard deviation of *Rhipicephalus microplus* larvae recovered from *Urochloa decumbens* pots 35 days after application of the following treatments: CTR 1: Control 1 - control aqueous Tween 80 at 1%; CTR 2: Control 2 - silicone at 0.01% + 1% aqueous Tween 80; CO: corn oil at 3%; CO + S: corn oil at 3% emulsified in Tween 80 at 1% and silicone at 0.01%; F: *C. javanica* at 10^8^ conidia/mL formulated in corn oil (3%), silicone (0.01%) and Tween 80 at 1%. Each group had ten pots, which received three fully engorged females of homogeneous weight. Means followed by the same letter do not differ significantly *P* < 0.05 (one-way ANOVA followed by a Tukey test)

Only the treatment with *C. javanica* at 1 × 10^8^ conidia/mL formulated in 3% corn oil, 0.01% silicone, and 1% Tween 80 resulted in a significant reduction in *R. microplus* larval recovery from *U. decumbens* pots with an average of 10.8 ± 15.2 larvae recovered (F_92.48_ = 4.49 and *P* < 0.0001) (Fig. 2). In comparison, the number of larvae recovered from the pots treated with Tween 80 1% was 1035 ± 221.9 and 807.2 ± 258.8 in the silicone (0.01%) + Tween 80 (1%). The corn oil (3%) yielded 811.3 ± 251.0 *R. microplus* larvae, while 790 ± 253 larvae were recovered from oil control 3% + silicone 0.01% + Tween 80 1%.

### Persistence of *Cordyceps javanica* in treated soil

**Fig. 3:**
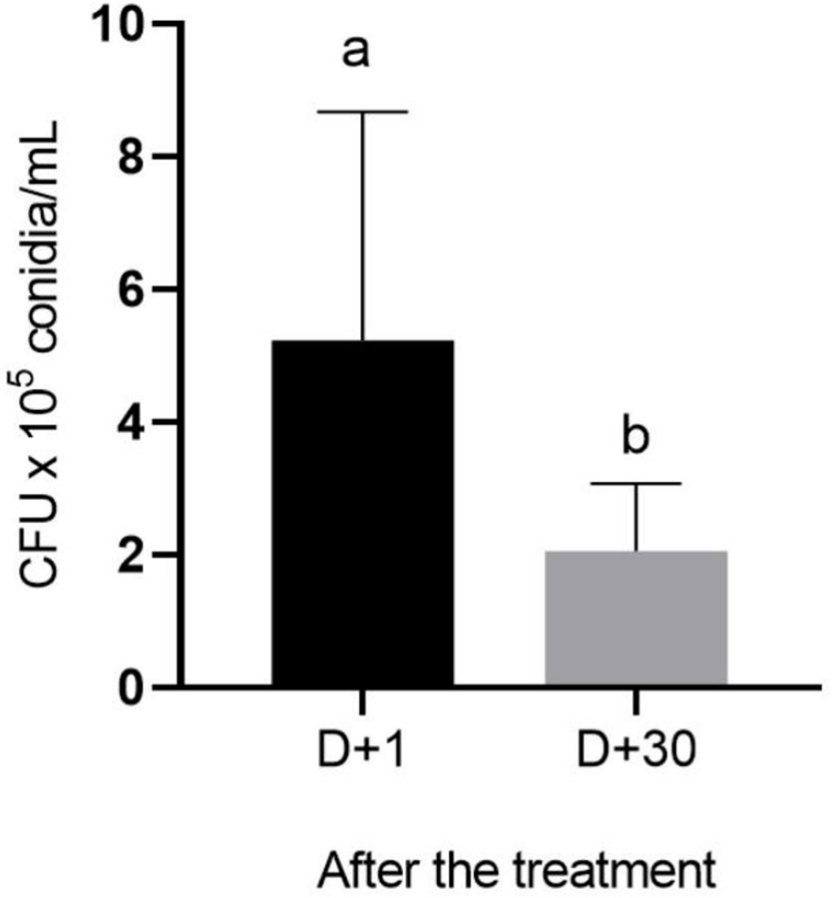
Mean number of colony forming units (CFU) of *Cordyceps javanica* recovered from the pots at 24 h (D + 1) and 30 days (D + 30) after application of the fungus at 10^8^ conidia/mL formulated in corn oil 3%, Tween 80 1% and silicone 0.01%. Different letters differ significantly considering *P* < 0.05 according to t test

Colonies of *C. javanica* were abundant in all soil samples collected from the fungus-treated group (fungus formulated 3%). *C. javanica* persisted in the soil up to 30 days after application, as evidenced by the number of viable conidia recovered from treated pots: 5.23 ± 1.09 × 10⁵ CFU/g of soil at 24 h and 2.06 ± 0.32 × 10⁵ CFU/g at 30 days post-treatment (t = 3.36; df = 19; *P* = 0.0083). No *Cordyceps*-colonies were.

detected in the soil from soil samples in control pots (i.e., tween 80 1%, silicone control, oil control 3%, and oil control 3% plus silicone) before or after the treatment (Fig. 3).

### Detection of silicone in soil samples by ATR-FTIR

**Fig. 4:**
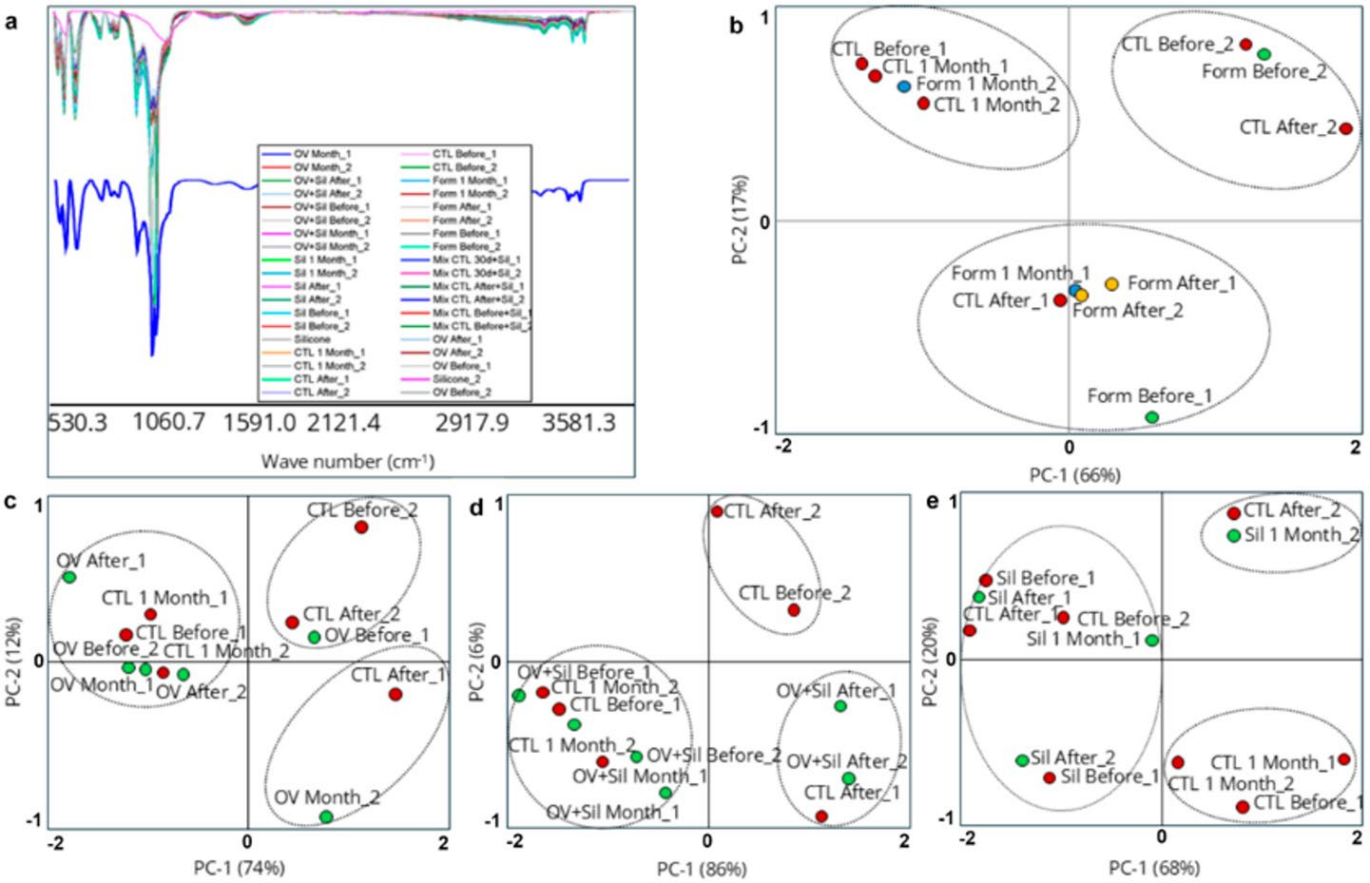
Chemometric analyses based on ATR-FTIR of soil treated with silicone 24 h before the treatments and 24 h and 30 days after the treatments. (**a**) Representation by line plot of all ATR-FTIR spectral and mean spectral (blue line) obtaining by chemometric analysis; (**b**) Graphics of PCA scores of CTR and FORM in the three different collection times; (**c**) Graphics of PCA scores of CTR and OV in the three different collection times; (**d**) Graphics of PCA scores of CTR and OV + SIL in the three different collection times; (**e**) Graphics of PCA scores of CTR and SIL in the three different collection times. (CTL) soil treated with Tween 80 (1%) aqueous suspension; (Sil) soil treated with aqueous emulsion of 0.01% silicone plus Tween 80 (1%); (OV) soil treated with aqueous emulsion of corn oil (3%) and Tween 80 (1%); (OV + Sil) soil treated with aqueous emulsion of corn oil (3%), Tween 80 (1%), and silicone (0.01%); (Form) soil treated with emulsion of conidia in corn oil (3%), silicone (0.01%), and Tween 80 (1%); (MIX) commercial standard silicone (Sigma) added to soil from aqueous control group (1:1); (Silicone) Standard silicone from Sigma. Before: soil collected 24 h before the treatment. After: soil collected 24 h after the treatment. Month: soil collected 30 days after the treatment

The ATR-FTIR spectra of soil samples collected (Table 6) confirmed a material with high organic complexity, rich in hydroxyl, carboxylic, and aromatic groups; containing polysaccharides, lignin, phenols, and humic/fulvic acids; and exhibiting secondary signs of associated inorganic salts or minerals (phosphates and silica, for example). This profile is fully consistent with a soil enriched with plant or humic material. The comparison between the infrared spectra of the treated soil samples and the standard silicone at 0.01% revealed striking differences in terms of chemical composition, according to the identified bands and their relative intensities (Fig. 4). The absence of fundamental silicone bands in the spectrum of treated soil samples, especially in the range 400–500 cm⁻¹ (Si–O–Si), 800–850 cm⁻¹ (CH₃–Si) and ~ 1100 cm⁻¹ (Si–O–Si stretching), reinforces that there was no clear spectroscopic evidence of the presence of silicone in the soil sample analyzed.

**Table 6:** Assignment of the main bands by spectral regions in the FTIR spectra of the soil after application of the different treatments

ν (cm⁻¹)	Probable Assignment	Observations
426–532	Inorganic vibrations (PO₄³⁻, Si–O, SO₄²⁻)	Clay minerals, phosphates or sulfates
688–794	C–H out of plane (aromatic) or rings	Lignin, phenolic compounds
912	C–O, C–H out of plane (aromatics), polysaccharides	Cellulose/lignin indicator
1003–1030	C–O, C–O–C, C–C stretches	Polysaccharides (cellulose, hemicellulose)
1115	C–O, C–N	Phenols, sugars, amines
1392–1412	Deformations CH₃/CH₂ ou COO⁻	Salts of organic acids (humic/fulvic acids)
1624	C=C aromatic or C=O (amides, carboxylic acids)	Lignin, humic compounds, adsorbed water
2795–2982	C–H stretching (aliphatic)	Presence of chains–CH₂–, –CH₃
3273–3693	Stretchings O–H / N–H	Alcohols, phenols, water, humic acids

The spectral range (ν, in cm⁻¹), the probable assignments of the main absorption bands and observations related to the soil constituents

### Application of chemometric techniques to evaluate possible changes in the soil pattern through the application of different formulations

The analysis of the grouped spectra (Fig. 4a) revealed that the commercial standard silicone added to the soil from the aqueous control group (MIX) was chemically distinct from the others, exhibiting bands characteristic of siloxane structures. In contrast, the soil samples, regardless of the treatment applied, displayed a complex chemical composition dominated by oxygenated organic compounds, primarily polysaccharides.

**Fig. 5:**
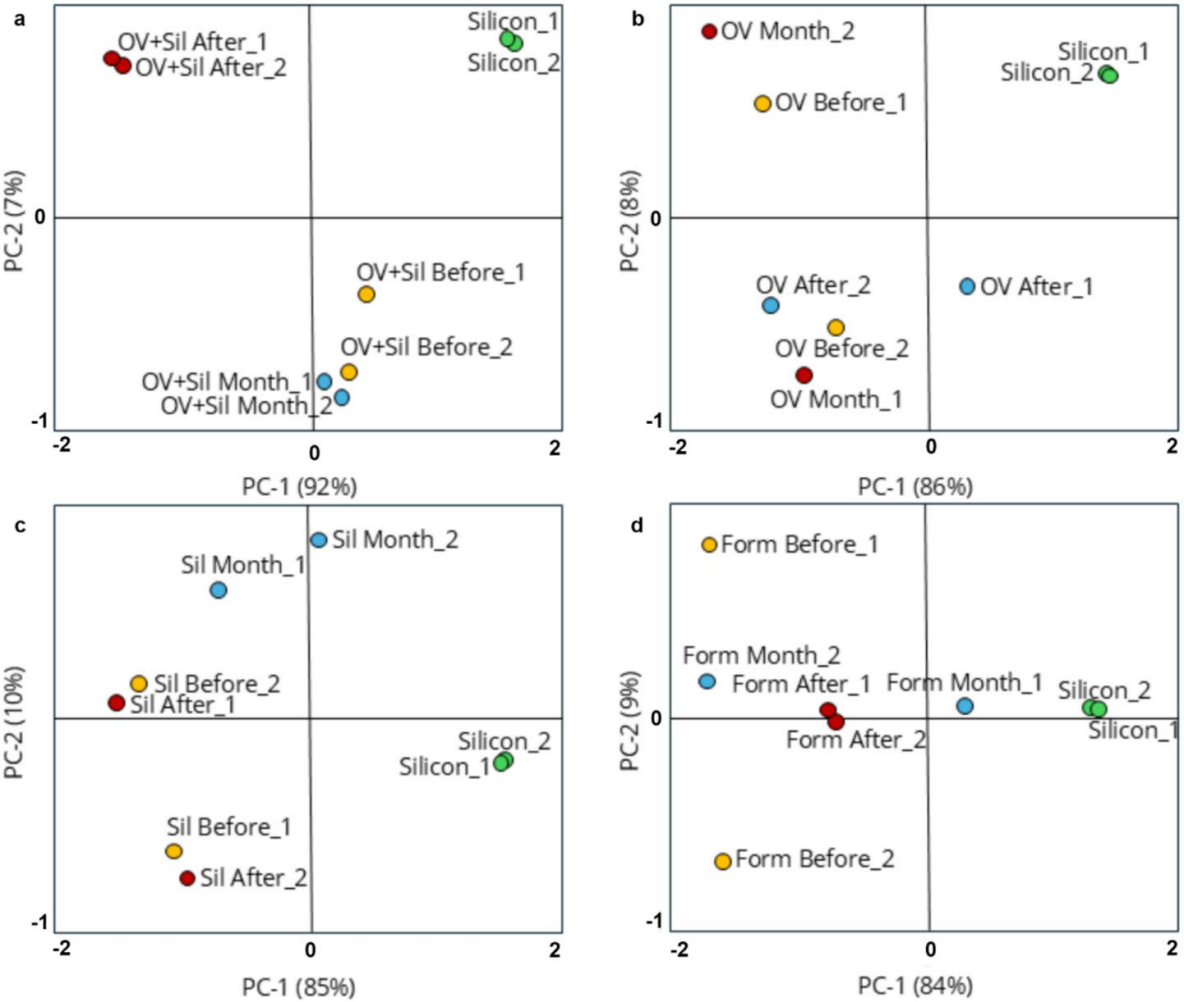
Principal Component Analysis (PCA) based on ATR-FTIR pure spectral data of standard silicone and soil samples treated with silicone. (**a**) Soil from OV + SIL compared to standard silicone; (**b**) Soil from OV compared to standard silicone; (**c**) Soil from SIL compared to standard silicone; (**d**) Soil from Form compared to standard silicone. (Sil) soil treated with aqueous emulsion of silicone (0.01% ) and Tween 80 (1%); (OV) soil treated with aqueous emulsion of corn oil (3%) and Tween 80 (1%); (OV + Sil) soil treated with aqueous emulsion of corn oil (3%), Tween 80 (1%), and silicone (0.01%); (Form) soil treated with emulsion of conidia in corn oil (3%), silicone (0.01%), and Tween 80 (1%); (Silicon) Standard silicone from Sigma. Before: soil collected 24 h before the treatment. After: soil collected 24 h after the treatment. Month: soil collected 30 days after the treatment

When compared directly with the standard silicone spectrum, silicone-containing soil samples were clearly separated, occupying isolated quadrants distant from the positive values of PC-1 (Fig. 5). In contrast, the treated and untreated soil samples remained grouped in distinct, but separate, regions. The chemical profile observed in the soil samples analyzed, regardless of the treatment applied, was entirely different from that of standard silicone (Fig. 5).

## Discussion

The initial steps in developing an oil-based formulation for an entomopathogenic fungus involve several critical evaluations and optimizations, including the selection of compatible oils to ensure that formulation adjuvants do not inhibit fungal growth or conidia germination. A second step involves conducting preliminary efficacy assays to confirm that the oil-based formulation retains the fungal isolate’s pathogenicity against the target pest. By beginning with these foundational steps, it is possible to identify early issues and build a robust baseline for subsequent field testing and develop formulations that are effective for tick control (Samish et al. 2014).

Adjuvants are commonly incorporated into fungal formulations to improve stability, adhesion, and overall efficacy (Rosado-Aguilar et al. 2017). Germination is a critical parameter to ensure that adjuvants do not compromise fungal viability, as conidia are the infective propagules responsible for initiating tick infection. In the present study, the germination of *C. javanica* conidia was assessed 24 h and 48 h after incubation with corn oil (1%, 3%, and 5% and silicone oil at 0.01%). Here, the statistical analysis confirmed that the presence of corn and silicone oils did not significantly affect conidial germination. Regardless of concentration, fungal viability remained high, indicating that both adjuvants were compatible with *C. javanica* and suitable for use in oil-based formulations. These findings ensure that the emulsified systems preserve the infective potential of conidia before application. Additionally, both corn and silicone oils are expected to exhibit favorable adhesion properties, potentially enhancing the fixation of conidia on the tick cuticle and thereby improving contact with the biological agent (Lei et al. 2023). Collectively, these findings suggest that the evaluated adjuvants are compatible with *C. javanica*, maintaining high germination rates while offering stability and adhesion benefits essential for successful formulation and field application.

After assessing the positive results of corn oil, silicone, and *C. javanica*’s biocompatibility, our study moved to the in vitro bioassays with tick females and larvae. Many studies have already tested the efficacy of entomopathogenic fungi against *R. microplus* larvae and engorged females (Camargo et al. 2012; Quinelato et al. 2012; Angelo et al. 2012; Nogueira et al. 2020), reporting high pathogenicity of a variety of fungal species and strains. In the present study, fungal suspension (no oil added) yielded low tick mortality, indicating the limited virulence of this unformulated fungal isolate under the tested conditions (Table 1; Fig. 1). Additionally, corn oil plus silicone with no fungus also caused low tick mortality (Table 1; Fig. 1).

The results of the in vitro bioassay with larvae and females highlighted the superior virulence of the fungus formulated with corn oil, compared to the control groups and unformulated fungal suspensions, regardless of conidial concentration. At both 10⁷ and 10⁸ conidia/mL, fungal emulsions demonstrated significantly higher mortality of *R. microplus* larvae and engorged females, with effectiveness increasing over time. As expected, among the tested treatments here, fungal formulations with corn oil (1, 3, and 5%) consistently outperformed the unformulated suspensions, achieving larval mortality rates of 100% 15 days after the treatment. Statistical testing confirmed a significant difference among treatments, indicating a synergistic interaction between the fungal conidia and the corn oil carrier (Table 2). This synergism likely results from improved adhesion and penetration of the fungus through the tick cuticle, which accelerates mortality and enhances virulence. These findings underscore the potential of corn oil-based formulations as effective adjuvants that enhance the speed and intensity of entomopathogenic fungal action against cattle tick, what can be explained by the increased adherence of conidia on the cuticle provided by the oil adjuvants present in the emulsions, which has a lipophilic property similar to the outermost part of the tick cuticle (Angelo et al. 2010; Camargo et al. 2012, 2014, 2016; Kaay and Hassan 2000).

Here, the oil percentage in the tested emulsions was similar [Barbieri et al. 2023 (2.5% mineral oil)] or much lower than in previous reports [Angelo et al. 2010 (15% mineral oil), Camargo et al. 2012 (10% mineral oil), Kaay and Hassan 2000 (15% peanut oil)]. Besides the lower oil concentration, the corn oil was chosen because vegetable oils are more readily biodegradable, reducing their environmental impact and the risk of long-term soil or water contamination (Siniawski et al. 2007; Rosado-Aguilar et al. 2017; Lee et al. 2022). To be precise, tests have indicated that vegetable oils undergo about 70–100% biodegradation in a period of 28 days (Aluyor et al. 2009). The lower hatching rates and egg production indices observed in the lab with the oil-based fungal formulations (3% or 5%) suggested that these treatments not only killed the ticks but also created a hostile environment that disrupted their life cycle.

The emulsion of 3% corn oil, 0.01% silicone, 1% Tween 80 and 10^8^ conidia/mL was used in the semifield tests because it yielded, in the laboratory tests, the best results (statistically not different from 5% corn oil), in this way, it was possible to use a lower percentage of oil, allowing a more rational and economical use of inputs without compromising the performance of tick control. The values for use in the field are the equivalent of 22 kg/ha of fungus, according to the applications in the pots of the semi-field test, which results in using a higher percentage of oil and a smaller quantity of fungal isolate, evidencing a better economic viability of the treatments. *U. decumbens* was selected as the forage in this trial to simulate typical Brazilian pasture conditions, as it is widely used in Brazilian cattle farming due to its adaptability to various biomes. It is worth noting that the seeds used were commercially coated with an antifungal agent, a common practice for phytosanitary protection. However, according to our results, the presence of the antifungal coating did not interfere with the action of *Cordyceps* on the tick present in the same pasture environment.

In the semi-field trial, the group treated with the fungal emulsion exhibited a drastically lower larval recovery in comparison to the control groups (Fig. 2). Drastic reduction of *R. microplus* larval outbreak in artificially infested grass was also reported with *Metarhizium* oil-in-water formulations (Mesquita et al. 2020; Marciano et al. 2020). Here, fungal suspensions were not tested in the semi-field trial due to their low efficacy in the laboratory tests. The significant reduction in larval recovery in the present study suggests a direct impact on the biological parameters and mortality of engorged females, reinforcing the acaricidal potential of this *Cordyceps* emulsion even under the effect of abiotic stressors such as high temperatures and solar UV radiation, which are deleterious for the fungus. Previous studies assessed the tolerance of fungal conidia in oil-in-water emulsions indicating higher tolerances of formulated fungi in comparison to unformulated (Paixão et al. 2017; Corval et al. 2020), suggesting the key oil role in protecting conidia viability against abiotic factors under field conditions (Mesquita et al. 2020; Marciano et al. 2020).

According to the literature, conidial survival under abiotic stressors can be enhanced by oil, contributing to greater fungal persistence in the soil (Marciano et al. 2020; Mesquita et al. 2020). Here, soil analysis of fungus-treated pots revealed the persistence of *C. javanica*, with 5.23 ± 1.09 × 10⁵ CFU/g recovered 24 h after application and 2.06 ± 0.32 × 10⁵ CFU/g 30 days later, indicating the fungus remained viable in the soil for an extended period following a single treatment. The decrease in CFU observed here (Fig. 3) can be attributed to the influence of abiotic factors, such as high temperature, low humidity, and high UV radiation, which often impact the viability of entomopathogenic fungi in the soil, but also may be attributed to the adaptability of the fungus in the soil environment (Mesquita et al. 2023). Despite this, here the total number of CFU per gram of soil, D + 30 after the treatment, was higher (i.e., 206.000 CFU/g of soil) than in other studies with similar conditions [i.e., Mesquita et al. (2020) ~ 50000 CFU/g of soil], suggesting that even though *C. javanica* conidia persistence 30 days after the treatment was lower than D + 1, the number of conidia was still enough to act against newly hatched *R. microplus* larvae.

As previously mentioned, the fungal emulsion was formulated with a minimal proportion of silicone (0.01%) in its composition. To support the development of a more sustainable bioproduct for controlling *R. microplus*, soil samples were analyzed over time to detect possible silicone residues. Thirty days after treatment, comparisons between standard silicone, silicone mixed with soil (MIX), and the treated soil samples revealed chemically distinct profiles (Fig. 4). These results suggest that either the silicone concentration in the treated soils was below the detection limit or that the organic components of the soil masked the siloxane signals, an inherent limitation of the FTIR technique used.

The application of chemometric techniques, specifically principal component analysis (PCA), allowed an in-depth evaluation of possible changes in the chemical pattern of the soil as a function of the different formulations applied. The data obtained demonstrate that the application of the different formulations tested (i.e., vegetable oil, vegetable oil with silicone at 0.01% and silicone alone at 0.01%) did not promote detectable changes in the overall chemical pattern of the soil samples, maintaining its composition dominated by organic compounds of plant origin.

The findings of the present study align with previous research emphasizing the importance of exploring combinations between entomopathogenic fungi and oils (Camargo et al. 2012; Marciano et al. 2020; Mesquita et al. 2020; Barbieri et al. 2023). The selection of corn oil as an adjuvant in the formulation is suggested to enhance conidial adhesion to the tick cuticle, while silicone improves the spreadability of the final product. Given that the use of *C. javanica* remains underexplored, this study represents a significant contribution to the state of the art in the biological control of hard ticks using bioinputs.

Although, as far as we know, the extent to which larval suppression in pasture directly translates into reduced tick infestation in cattle has not yet been studied, effective tick control necessarily involves interventions targeting the environment, as much of the tick life cycle occurs off host. From an integrated tick management perspective, the application of entomopathogenic fungi to pasture soil represents a preventive strategy aimed at reducing larval pressure before host infestation. This approach aligns with integrated management frameworks that combine biological control, environmental interventions, and rational acaricide use to achieve sustainable control and delay resistance development (Lagunes-Quintanilla et al. 2024). By lowering the abundance of larvae in pasture, soil-applied fungal biocontrol may reduce the need for treatments applied directly to cattle, contributing to improved animal welfare and reduced chemical inputs.

## Conclusion

The tests performed, including in vitro and semi-field assays, demonstrated the high effectiveness of *C. javanica* formulated in corn oil against *R. microplus*, highlighting the importance of the oils added in the acaricidal effect of the formulations.

## Data Availability

The data used to support the findings of this study are available from the corresponding author on reasonable request.
